# Asymmetric flow field-flow fractionation as a multifunctional technique for the characterization of polymeric nanocarriers

**DOI:** 10.1007/s13346-021-00918-5

**Published:** 2021-01-31

**Authors:** Federico Quattrini, Germán Berrecoso, José Crecente-Campo, María José Alonso

**Affiliations:** 1 Center for Research in Molecular Medicine and Chronic Diseases, Singular Research Centers, 15782 Santiago de Compostela, Spain; 2 Instituto de Investigación Sanitaria de Santiago de Compostela (IDIS), IDIS Research Institute, 15706 Santiago de Compostela, Spain; 3 Department of Pharmacy and Pharmaceutical Technology, School of Pharmacy, Universidade de Santiago de Compostela, 15782 Santiago de Compostela, Spain

**Keywords:** Polymeric nanoparticles, Asymmetric flow field-flow fractionation, Biocorona, Drug delivery, Multiangle light scattering

## Abstract

The importance of polymeric nanocarriers in the field of drug delivery is ever-increasing, and the accurate characterization of their properties is paramount to understand and predict their behavior. Asymmetric flow field-flow fractionation (AF4) is a fractionation technique that has gained considerable attention for its gentle separation conditions, broad working range, and versatility. AF4 can be hyphenated to a plurality of concentration and size detectors, thus permitting the analysis of the multifunctionality of nanomaterials. Despite this potential, the practical information that can be retrieved by AF4 and its possible applications are still rather unfamiliar to the pharmaceutical scientist. This review was conceived as a primer that clearly states the “do’s and don’ts” about AF4 applied to the characterization of polymeric nanocarriers. Aside from size characterization, AF4 can be beneficial during formulation optimization, for drug loading and drug release determination and for the study of interactions among biomaterials. It will focus mainly on the advances made in the last 5 years, as well as indicating the problematics on the consensus, which have not been reached yet. Methodological recommendations for several case studies will be also included.

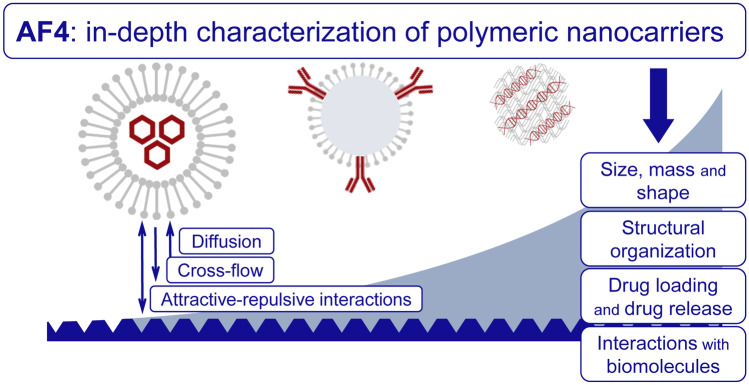

## Introduction

In the last decades, nanotechnology has assumed a central role in the field of drug delivery [1, 2]. Nanotechnology can help complex drugs to overcome biological barriers, facilitate their solubilization, reduce their toxicity, and improve their targeting to specific tissues and cells. While the application of iron oxide nanoparticles for the treatment of iron deficiency anemia can be traced back to the early 20^th^ century [3], the burst of interest in the field of nanomedicine has often been associated to the approval of Doxil® in 1995. Now, 25 years later, more than 50 nanomedicines have been made commercially available [4], and several hundreds are undergoing clinical trials.

A vast array of nanoparticles of diverse chemical composition and morphology is now available, from inorganic nanoparticles to solid lipid nanoparticles, to proteins-based nanoparticles to a variety of polymer nanostructural assemblies. The last ones are gaining particular attention, thanks to their variety and versatility, as well as the ability to accommodate multiple kinds of drugs and to be functionalized [5]. They are present in formats such as nanoparticles, polymer complexes, micelles, nanocapsules, nanogels, and dendrimers-based particles [6–8]. Despite their intrinsic diversity, their properties and efficacy are described by a common set of parameters: their size, stability in biological media, drug loading, and kinetics of drug release. The study and quantification of these parameters is not an easy task, since these nanocarriers are often heterogeneous in their composition, size, shape, and physicochemical properties. In addition, their behavior in biological media is often unpredictable, given the complexity of the transformations and re-organizations that occur because of the interactions with biological molecules, such as the formation of the protein corona. Their characterization requires techniques that can handle their heterogenicity.

The family of techniques for the fractionation of nanomaterials known as field-flow fractionation (FFF) is relatively recent. Its first applications date back to the seventies but its most successful incarnation, asymmetric flow field-flow fractionation (AF4), was commercialized only in the late nineties. While FFF techniques are diverse among each other, each of them being more suitable for specific purposes, they all are based on the same principle: solutes fractionate among layers of a continuous flow under the influence of an external field. The main advantages of these techniques are a wider operational range, superior sizing accuracy, compatibility with numerous types of detectors, and greater separation power, especially for polydisperse samples [9, 10]. In the last few years, the application of FFF to the field of nanotechnology for medical applications started being recognized by the regulatory agencies. The first ISO standard for the analysis of nano-objects using AF4 and Centrifugal FFF (ISO/TS 21362:2018) was published in 2018. In the same year, the EUNCL, in collaboration with the NCI-NCL, developed a standard operating procedure to determine the size distribution of nanoparticles for medical applications by coupling AF4 with online size measurement [11].

Despite AF4 being an interesting candidate for the characterization of nanocarriers, its applications have not been as extensive as expected, especially if compared with other chromatographic techniques. Aside from some practical reasons, such as the high cost of the equipment and the need for trained personnel, one of the causes behind its limited popularity is that its potential applications are still relatively overlooked. In most cases, AF4 is employed simply as a sizing technique and in this way, given its cost and complexity, is clearly underused. Similarly, the number of reliable, generally applicable protocols is still rather limited. While the necessity of a case-by-case approach to method development is inherent to the technique, some general guidelines can be extracted from the literature.

In the last 5 years, several works have reviewed the main developments in the biomedical applications of FFF. Back in 2014, the work of Wagner et al. [12] introduced the applications of AF4 to nanomedicine, comparing it with other fractionation techniques and stating its advantages and pitfalls. The same year Zattoni et al*.* [13] focused specifically on the use of AF4 for the characterization of several kinds of nanoparticles for drug delivery, in particular liposomes, organic polymers, and virus-like particles. Then, Malik and Pasch [14] reported the applications to polymer analysis, and Zhang et al. [15] summarized the theoretical principles of several members of the FFF family. Subsequent updates covered different kinds of nanomaterials [16, 17] including virus-like particles [18], and the inherent limitations associated with the membranes used in AF4 have also been reported [19].

Here we would like to take a different, more down-to-earth approach. Our objective is to provide the reader with a view of the kinds of information about polymeric nanocarriers retrievable via AF4. To do so, after describing some fundamentals and practical issues of AF4, we structured this review as a series of answers to the following questions, all of them related to the formulation and characterization of nanomedicines: (i) can AF4 determine the size distribution, and other physical properties, of heterogeneous polymeric colloidal systems?; (ii) can AF4 offer insights into the structural organization of the components of polymeric nanocarriers?; (iii) can AF4 provide information about the attachment of drug molecules to the nanocarriers and their release?; and (iv) what kind of information can AF4 provide about the formation of the protein corona?

## The FFF Family

**Fig. 1:**
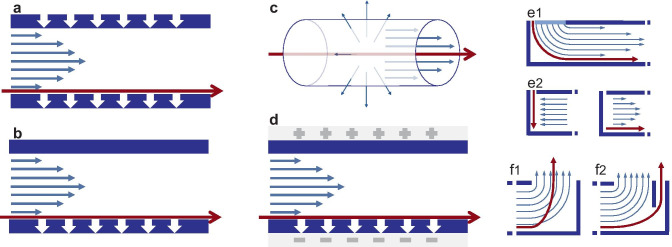
Types of channel for FlFFF. Symmetric flow FFF **a**; asymmetric flow FFF **b**; hollow fiber flow FFF **c**; electrical asymmetric flow FFF **d**; stopless frit-inlet FFF **e1** and traditional FFF with focusing and elution step **e2**; traditional outlet **f1** and slot-outlet **f2**

Field-flow fractionation is a family of techniques, closely related to chromatography, specifically designed for the fractionation of materials of nanometric size. Unlike chromatography, in FFF techniques there is no stationary phase. Instead, the separation of the solutes takes place in a ribbon-like channel, built so as to stress the laminar flow of the eluent, which will move in layers of increasingly higher speed, going from the walls to the center of the channel. In flow FFF (FlFFF) a secondary flow of eluent pushes the solutes towards one of the walls of the channel. The different variants of this technique are illustrated in Fig. 1. In its most common design, asymmetric flow FFF, this is accomplished by having one of the channel walls replaced by a filtration membrane, through which the eluent is pumped out. As a reaction, the solutes will diffuse in the opposite direction, at different velocities related to their size, finally partitioning among the layers of the eluent.

A variation of AF4 is hollow fiber flow FFF (HF5), where the channel is replaced by a tubular membrane with porous walls, which allows to reduce sample dilution up to an order of magnitude and increases separation efficiency [20]. A further variant of FlFFF is the frit-inlet flow field-flow fractionation [21]. In traditional FFF the fractionation consists of two steps, the focusing, and the elution. During the focusing step, an additional flow keeps the sample at the head of the channel, giving the solutes enough time to reach a steady-state. Then, in the elution step, the solutes are allowed to move along the channel, resulting in the size-based separation. The focusing step, though essential for the fractionation, may also result in undesired sample-membrane interactions and consequent sample loss. The need for the focusing step is avoided in frit-inlet flow FFF, as the sample is compressed towards the head of the channel by a continuous flow of eluent at high speed. Another modification to the classic channel, the so-called slot-outlet technology, allows to reduce sample dilution, lowering the limit of detection up to an order of magnitude [22]. The most recent technique in the family is the electrical asymmetric flow FFF (EAF4) where, in addition to the secondary flow, an electric field is generated across the channel. In EAF4 the separation is ruled both by size and electrophoretic mobility, which allows the simultaneous determination of size and particle surface charge [23].

Other FFF techniques have been developed along the years and are now commercially available, such as thermal FFF (ThFFF) [24], electrical FFF (ElFFF) [25], or sedimentation/centrifugal FFF (SdFFF or CF3) [26]. SdFFF separates nanomaterials according to both their density and size, and some of its applications to nanocarriers for drug delivery will be mentioned later.

The miniaturization of FFF techniques is also of particular interest, from the perspective of lower sample requirement, better resolution, lower carrier volumes, and faster analysis time. While the miniaturization of FlFFF has provided some benefits, such as lower limits of detection [27], it was quite a game-changer in the case of ThFFF and ElFFF [28], allowing much lower power consumption and improved operational flexibility and de facto opening a new field of possibilities.

### Principles of FFF

Focusing on the physical principles behind the fractionation can explain one of the main advantages of FFF: particle size can be directly computed from known or easily measurable values, without the need of a size detector or external calibration.

**Fig. 2:**
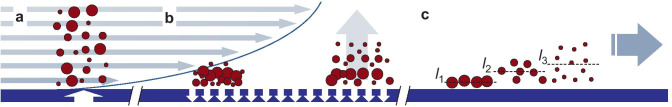
Simplified scheme of an AF4 channel operating in the normal mode. Sample injection **a**; sample focusing **b**; separation of the particle “clouds” with characteristics elevation *l*_n_ **c**

As anticipated, the solutes in the channel are subjected to both an externally induced flow and a diffusion flow. As shown in Fig. 2, the effect of these two opposite flows forces the particles to spread across the channel radius, forming a “cloud” defined by its mean layer thickness *l* [29]. In FlFFF the value of *l* at steady-state will depend uniquely on the diffusion coefficient and, through the Stokes-Einstein equation, on the hydrodynamic radius of the particles [16]. As a first approximation, the hydrodynamic radius of a particle can be found with the following equation:

1 $${r}_{h}=\frac{1}{6l}\frac{kT{V}^{0}}{\pi \eta w\dot{V}}=\frac{{t}_{r}}{{t}^{0}}\frac{kT{V}^{0}}{\pi \eta {w}^{2}\dot{V}}$$

where *t*_r_ is the retention time of the solute, *t*^0^ is the time needed for the eluent to pass through the channel (retention time of the void volume peak), *k* is the Boltzmann constant, *T* is the absolute temperature, *V*^0^ is the volume of the channel, *η* is the viscosity of the medium, *w* is the channel thickness, and $\dot{V}$ is the cross-flow rate. It is easy to see that for constant values of $\dot{V}$, there is a linear relationship between particle size and retention time.

Several guidelines have been developed for the determination of the values of *V*^0^, *t*^0^, and *w* for a specific channel configuration [30]. For most practical purposes, the equation can be treated as a semiempirical law; the relationship between the retention time *t*_r_ and the particle size can be found by building a calibration line running standards of known size.

Exceptions to the theory are not infrequent and, in particular cases, the solutes can obey to different mechanisms. For particles of micrometric size, hydrodynamic forces or steric interactions can have a larger impact than diffusion. In this case, called steric/hyperlayer or “reversed” mode of elution, retention time is inversely proportional to particle size, rather than directly as in the “normal” mode of elution described above. Instances of “reversed” mode can occur also with nanometric objects, especially for high values of the cross-flow, and can hinder the interpretation of the fractogram [31–33].

Deviation from the theory aside, in most practical application FFF, the sizing is carried out by coupling a light-scattering detector to the channel, instead of relying exclusively on the analysis of the retention time. There are several reasons for this: first, real samples often require using decaying cross-flow to optimize the fractionation, whereas the relationship between *l* and the retention time is linear only for constant cross-flow. Analytical solutions for the retention time with exponential decay of cross-flow rate are available [34], as well as numerical solutions for the more general cases [35], yet they are of difficult implementation and limited use for the experimentalist. Second, the latex standards normally required for size calibration to be fractionated using detergent-enriched eluents (common choices are 0.2% Novachem [36], 0.02% FL-70 [37], 0.1% SDS [38], or similar) to prevent their aggregation, but these conditions may not be suitable for the samples [39]. And lastly and most importantly, the information provided by the online light-scattering detector complements, rather than substitutes, the one obtained from the retention time, as it will be shown in the following sections.

AF4 is the most readily available technique among the FFFs and the most common in pharmaceutical science. The reason behind its success are manifold: first of all, the mobile phase can be chosen freely, allowing the study of biological samples in physiological conditions (ElFFF, on the contrary, requires eluents with low ionic strength, and ThFFF mostly works with organic eluents); the separation is based exclusively on size; its setup is simpler and more robust than the other techniques; part of the channel (or the whole channel, in the case of HF5) is disposable, reducing the risk of cross-contamination. Specific applications of the other members of the FFF family in the field of nanomedicine will be mentioned later in the text and are summarized in Table 1.

**Table 1:** The FFF techniques and their possible applications to nanomaterials for drug delivery

Technique	Separation principle	Separation depends on	Eluent	Applications to drug delivery systems	Ref
ThFFF	Temperature gradient	*D*, *D*_T_	Mostly organic (few examples in aqueous buffer)	Separate polymeric micelles according to size and polymer composition	[191, 192]
GrFFF and SdFFF (CF3)	Gravitational force	*m*, *δ*, *D*	Mostly free (more than FlFFF, since there is no membrane)	Separate particles from larger aggregates, “differential FFF”	[76, 128][118]
ElFFF	Electrical force	*μ*, *D*	Deionized water	Separate particles with different surface functionalization and *z*-potential	[193]
EAF4	Electrical force + secondary eluent flow	*μ*, *D*	Mostly free	Separate particles with different surface functionalization and *z*-potential	[75]
FlFFF (AF4 or HF5)	Secondary eluent flow	*D*	Mostly free	See main text	–-

*D* diffusion coefficient, *D*_*T*_ thermal diffusion coefficient, *m* molar mass, *δ* density, *μ* electrophoretic mobility

## Method development: analysis of nanocarriers

Excellent advice for good method development practice can be found in several works [18, 30, 40]. In general, nanocarrier formulations are usually composed of a main population, along with reagents in excess or non-encapsulated drug. A requisite for adequate fractionation and quantification is to avoid non-specific particle-membrane and particle-particle interactions. A passing knowledge of these phenomena can be helpful for a rational experimental design.

### Particle-membrane interactions

**Fig. 3:**
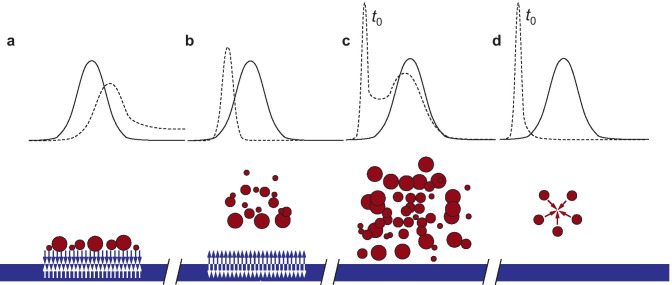
Ideal (solid line) vs. non-ideal conditions (dashed line). Particle-membrane attraction: sample loss, shift to longer retention time, peak tailing **a**; particle-membrane repulsion: poor separation, shift to shorter retention time **b**; overloading: large void volume peak, concentration-dependent retention time **c**; particle–particle attraction: sample aggregation, steric/hyperlayer elution **d**

As shown in Fig. 3, membrane-particle interactions, which can be of electrostatic, hydrophobic, or steric nature, may affect sample recovery and retention time as well [41, 42]. Sample loss tends to diminish after a few fractionations, and freshly changed membranes are sometimes preconditioned before the analyses by performing sacrificial fractionations of BSA to saturate their binding sites [43, 44]. The most common material for membranes, at least for the analysis of biological samples, is regenerated cellulose (RC) [45]. RC has pKa ∼ 3.5 [46, 47] and has a negative charge at physiological pH. Electrostatic forces are of special importance in determining solute-membrane interactions and should be controlled by choosing the appropriate eluent [46] or, if available, the appropriate membrane. Positively charged materials, for example, are better fractionated on membranes with lower surface charge, such as amphiphilic membranes [48]. While attractive interactions have to be avoided, strong particle-membrane charge repulsions are not recommended either, because the solutes will not get close enough to the membrane, negatively affecting the quality of the separation [12].

Hydrophobic interactions can also result in solutes adsorption on the membrane. They operate at shorter distances than the electrostatic force and, thus, they are more relevant for smaller particles that can enter the membrane pores. Polyethersulfone membranes are more prone than cellulose to this kind of interactions [49].

In addition to surface charge and material, membranes are defined by their porosity. Sample loss may take place even when the membrane molecular-weight cut-off (MWCO) is lower than particle size, as also other factors describe the surface properties of the membrane. In addition to membrane MWCO, particle shape, membrane pore density, and cross-flow intensity also play a role in determining sample recovery [50].

Permanent functionalization of the membrane by chemical or physical means could be a further way to improve separation and at the same time reduce undesired interactions. To this purpose, it has lately been proposed the use of microstructured filtration membranes that present slanted grooves on their surface. The grooves not only increase sample retention and resolution [51] but also permit to carry out a two-dimensional size-based separation, as the solutes are subjected to size-related lateral displacement [52].

Even though the mechanism of fractionation in the AF4 channel is well described, the effects of particle-membrane interactions are still mostly unknown and a systematic characterization (pH-dependent surface charge, effective pore size, lipophilic interactions) of the commercially available membranes is still lacking. Fortunately, several important steps in this direction have been taken in the last few years [19, 49].

### Particle-particle interactions and overloading

During the focusing step illustrated in Fig. 2, the solutes are concentrated in a thin layer of solution, which may result in intensified particle-particle interactions. Interactions are both attractive or repulsive and are heavily influenced by the composition of the eluent, its ionic strength, and concentration of detergents. They commonly take place in cases of sample overloading, together with particle-membrane interactions. Depending on the kind of material and eluent composition, overloading may result in peak shift at shorter or longer times, fronting or tailing, larger void volume peak, and incomplete separation. Concentration-dependent retention time is a reliable and easy-to-check indicator of overloading [49, 53, 54]. Biopolymers of high molecular mass are especially prone to overloading [55, 56]. Additional ways to prevent overloading without having to reduce the amount of sample injected are to reduce the cross-flows [30], optimize the flows in order to focus the sample in the region of the channel with larger breadth [57], or directly use larger channels [58].

### Towards a multivariate experimental design

The application of multivariate analysis in method development has become a staple of chromatographic techniques such as high-performance liquid chromatography (HPLC) for being robust, efficient, and cost-effective [59]. With AF4 becoming increasingly common there have been several attempts at applying multivariate design to the development of methods for the fractionation of proteins and human serum [60], TiO_2_ nanoparticles [61], and Ag nanoparticles in natural waters [62]. To the best of our knowledge, it has not been applied yet to the fractionation of nanoformulations. In multivariate experimental design, instead of optimizing one parameter at a time (say, first optimize the eluent composition, then the amount of sample injected, etc.), all the parameters are changed together following a statistical design that allows a better representation of the whole process, also taking into account the combined effect of the parameters. The results obtained in the cited studies are in agreement with the theory and the common knowledge of AF4, for example, restating the importance of the eluent composition and cross-flow over most of the other parameters [62]. Multivariate design, while comports some additional effort, may be beneficial, especially for routine applications.

## Determine size distribution and physical properties of nanocarriers

### Particle size

Generally, the diameter of nanomaterials for drug delivery falls in the range between *d* = 10 and 200 nm, whose limits are roughly defined by the molecular filtration size cut-off and the reticuloendothelial system uptake cut-off [63]. The size distribution of the nanocarriers is one of the primary factors in determining cell uptake, entry into tumors, as well as their maximum drug loading and functionalization capacity. So, aside from being accurate, a reliable sizing technique will also be able to cover a size range spanning over 2–3 orders of magnitudes (Table 2).

**Table 2:** Comparison of the principal sizing techniques used for the characterization of polymeric nanocarriers

Technique	Size range	Information retrieved	Pros	Cons
DLS	*d*: ~ 10–1000 nm	Hydrodynamic radius Polydispersity index	Fast. Large size range	Highly size-biased
NTA	*d*: ~ 50–1000 nm	Hydrodynamic radius Polydispersity index Particle number	Particle counting. Less size-biased than batch DLS	Higher low-size limit than DLS. Difficult to couple to fractionation techniques
MALS	*r*_g_: ~ 10–500 nm Mass: 1–10^6^ kDa (with Rayleigh-Gans approximation)	Gyration radius. Molecular mass	Only technique that can assess mass and geometric radius	Requires monodisperse samples (must be coupled to SEC or AF4)
SEC	*d*: ~ 0.1 to ~ 50 nm	Hydrodynamic radius	Sample fractionation. Can handle small molecules	Limited size range. Non-specific interactions with the resin
AF4	*d*: ~ 1–1000 nm	Hydrodynamic radius	Sample fractionation. Wide size range. Free eluent	Higher low-size limit than SEC. Non-specific interactions with the membrane

Among the batch techniques used for the sizing of nanocarriers, the most common is probably nanoparticle tracking analysis (DLS or QELS), where the hydrodynamic size of the particles is computed from their Brownian diffusion. Batch DLS allows fast and easy-to-perform analyses, but it is strongly biased towards larger objects, a pitfall already underlined by regulatory bodies [64]. Another light scattering technique of recent design, nanoparticle tracking analysis (NTA), generally offers a more complete characterization than DLS, measuring both size and number of particles. In comparison to DLS, it is much less size-biased [65], yet it has higher lower-size limit in comparison to DLS (*d* > 40–50 nm), which does not allow its application to proteins or small nanocarriers [66].

AF4 is often compared with size-exclusion chromatography (SEC), as they are fractionation and sizing techniques that operate over a similar size range and, in many cases, can be applied almost interchangeably, providing results of similar quality [67, 68]. Though similar, they have their own peculiarity and limitations that make them more suitable for specific purposes. First, AF4 has a much broader operational range that allows the separation of materials spanning over at least two orders of magnitude in a single run, while SEC would require using stationary phases of different pore sizes. For the same reason, AF4 is less susceptible to fouling by particulates, and sample filtration is required only to remove macroscopic particles [69]. That said, SEC can handle particles in the range *d* = 1–10 nm more efficiently than AF4, as it is not limited by the MWCO of the filtration membrane. This, and the fact that it is more suited than AF4 to working with organic solvents, makes SEC a better choice for polymer characterization [70]. The presence of the stationary phase may comport lower recoveries than AF4, although this is heavily influenced by the material analyzed and the fractionation conditions [36].

As anticipated, particle size can be measured by AF4 in two ways: or directly from the retention time, by applying the theoretical considerations discussed before, or by coupling it to a size detector. The most common choice is multi angle laser light scattering (MALLS or MALS), which will be discussed in detail in the following paragraph. DLS can be coupled to AF4 as well, and the size-based fractionation greatly enhances its performance, preventing the bias towards larger sizes [71]. The user must be aware that coupling DLS to AF4 for online sizing does not come without drawbacks, since the particles will be affected not only by Brownian motion, as in batch DLS, but also by the eluent flow, which interferes with size determination. This discrepancy increases with particle size and detector-flow velocity, and it was reported to be as high as 50%, independently from the sample material [72]. It is good practice to check for variations in the size measured by online DLS at different flow rates [39]. Nevertheless, in most of the literature here reviewed the results from online DLS are assumed to be independent of the flow rate and could be trusted without the need for further corrections.

Coupling NTA and AF4 could provide information about particles number, concentration, and size in a single run, as well as increasing the resolution power of both techniques. The main challenge is that NTA employs much lower flow-rates than AF4 and therefore cannot be directly coupled to AF4. Several ways to circumvent this problem have been found: to use a switching valve to periodically divert aliquots of the fluid to the NTA chamber [73], or to split the main stream into multiple flow path by using a splitter manifold connection [74] or again by using the slot-outlet technology [75].

As much as the fractionation improves particle sizing with respect to batch techniques, it may present some rare drawbacks. For example, batch DLS was able to detect the aggregates present in a formulation of poly(lactic-co-glycolic acid) (PLGA) nanoparticles, while said aggregates disappeared when the formulation was analyzed by SdFFF due to the spatial separation among solutes [76]. In a similar fashion, excessively high flows could induce particle disruption due to shear forces [77].

The reason why MALS is often the size detector of preference is that it provides information complementary to the one of AF4 or DLS. Rather than the hydrodynamic radius, it measures the molar mass and the radius of gyration (*r*_g_, or root-mean-square radius) of the solutes. The radius of gyration is related to the geometric radius and the distribution of the mass in the particles. In addition, measuring the molar mass can prove particularly meaningful for materials with a precisely defined structure, where it can provide information about the number of subunits or the stoichiometry of a complex. For example, AF4-MALS can ascertain how chemical functionalization affects the aggregation number of nanogels [78] or polymeric micelles [79], or assess the stoichiometry of polymeric carriers/nanocomplexes [80, 81]. On the downside, MALS needs purely monodisperse samples, making the coupling to a fractionation technique necessary. In addition, size determination is less direct than with other techniques, as it requires fitting the scattering data obtained by the detector with a suitable model. A number of extrapolation methods are provided by commercial software, some based only on the theory of light scattering, others based on further assumptions on the geometry of the particles. The choice of the correct extrapolation method is not always straightforward and may lead to substantial estimation errors [82]. A systematic study of the applicability of each method can be found in Andersson et al. [83]. These extrapolation methods are generally used for particles with *r*_g_ < 50 nm, while the scattering data from larger objects are more accurately fitted by models that take into account their geometry (such as spherical or random coil—for a comprehensive list v. Wyatt [84]). This poses a problem, since particle geometry may be unknown, and different fitting models can give substantially different results.

The operational range of the technique is another factor to be taken into account when evaluating the results. The operational range of MALS normally lies between *r*_g_ = 10 and 500 nm, covering the size range of most nanomaterials employed in drug delivery. There are some exceptions, though: for example, single- and multi-walled nanotubes, which have one dimension bordering on the micrometric range, have found several applications in the field of nanomedicine [85]. In general, objects with very high aspect ratio present challenges not only for MALS characterization, but for AF4 fractionation as well [86–89]. For example, carbon-nanotube-loaded poly-*ε*-caprolactone nanoemulsions, which assumed a rod-like shape, were found to elute much sooner than blank nanoemulsions, which retained their original spherical shape [89].

### Particle shape

The data retrieved by AF4-DLS and AF4-MALS can provide further insight into the shape and structure of the nanocarriers. It is known that the ratio between the radius of gyration *r*_g_ and the hydrodynamic radius *r*_h_, the so-called shape factor, is an important descriptor of particle shape. It can be demonstrated that homogeneous hard spheres have a shape factor close to 0.778, while values > 1 are indicator of particles with high aspect ratio. Values of the shape factor < 0.7 suggest particles with a dense core surrounded by a lighter shell [90]. Sometimes determining the shape of a particle only on the basis of the shape factor may lead to results of difficult physical interpretation [91, 92]. Potential sources of error on the *r*_g_ can be the coelution of materials of similar hydrodynamic size, but different shape or mass distribution, as a nanoparticle and a protein [93].

Another parameter representative of sample morphology is the fractal dimension (*D*_m_). The aggregation of colloidal suspensions produces fractal objects, for which molar mass *m* and size *r*_g_ are related by an expression of this kind:

2 $$m={{r}_{g}}^{{D}_{m}}$$

*D*_m_ can be related to the compactness of the system: high values (> 2.5) correspond to dense spherical particles, while more branched structures are associated with lower values and a value of 1 indicates rod-like particles. This kind of analysis can turn useful during carrier formulation to detect the formation of aggregates. As an example, a reported work [94] aimed to compare particles of chitosan and hyaluronate formulated in two alternative ways, by simply mixing the two polymers or by adding TPP as a template to promote the gelification of chitosan before adding hyaluronic acid. While both kinds of particles displayed values of *D*_m_ > 2, typical for a spherical shape, those obtained by template addition were significantly smaller, suggesting that the hyaluronate forms a less dense shell around the chitosan core.

Shape determination plays an important role in quality control because it permits to identify the presence of aggregates, a parameter with many consequences on formulation stability, use predictability, and drug potency [95, 96]. Techniques that provide information about size but not shape, such as DLS or NTA, are not able to distinguish between aggregates of small particles and larger particles, an important difference when it comes to optimizing the formulation process. In AF4-MALS-DLS aggregates can be differentiated from single units not only by their size but also by the higher value of the shape factor, which corresponds to less dense structures [97]. Similarly, AF4-MALS-DLS showed how the incorporation of paclitaxel in polymeric micelles by the post-insertion method resulted in the formation of micelles clusters. The presence of the clusters would have interfered with batch DLS measurement, even if the concentration and size of the single micelles did not change after the insertion, again demonstrating the usefulness of AF4 in quality control [98].

### Particle surface charge

A further parameter that defines the behavior of a nanocarrier, from the formation of the protein corona to its internalization, is the surface charge. The measurement of the *z*-potential, the charge at the solid/water interface, is normally performed by electrophoretic light scattering (ELS) [99], a technique not compatible with online detection. In general, the characterization of particle surface charge is carried out on fractions collected after AF4 separation. As an example, the offline hyphenation of AF4 and capillary electrophoresis has been applied to differentiate plain and carboxylated polystyrene nanoparticles [100, 101].

On the other hand, EAF4, the most recent variation of AF4, is a particularly promising option for the online estimation of the *z*-potential. In EAF4 the separation is mainly driven by the flow force field, as in traditional AF4, while the superimposed electric field causes a minor shift in the retention time, which is related to the electrophoretic mobility of the solute. A major advantage of EAF4 over the traditional electrical FFF is that it is not limited to eluents at low ionic strength, so allowing it to work in physiological conditions. EAF4 was recently used to fractionate liposomal formulations of doxorubicin incubated in phosphate buffer saline (PBS) and Dulbecco’s modified Eagle medium (DMEM) cell culture buffer. The size of the liposomes did not change after incubation in the protein-enriched buffer, whereas their *z*-potential underwent a 10-mV increase, suggesting that their surface properties changed even if a proper protein corona did not form [75].

## Study the structural organization of the nanocarriers

### Pre-formulation studies: characterization of biomaterials

A major cause of irreproducibility in the formulation of nanosystems for drug delivery is the frequently observed lack of standardization of the starting materials, and their poor characterization [102]. To this purpose, AF4 can be used to characterize the polymers constituting the “building blocks” of the nanocarriers, even before the synthesis and formulation steps. For example, AF4-UV-MALS was able to determine the molecular mass distribution of samples of industrial gelatin, which could be correlated to their physicochemical properties, gel strength, and viscosity [103]. Similarly, starches grafted with ether or acetyl groups for bioplastic applications were compared by AF4-RI-MALS. The molar mass of the starch was found to change depending on the functional group [104].

On the other hand, thermostatic AF4 channels are commercially available and are an efficient tool for the characterization of temperature-responsive materials. Derivatives of poly(*N*-isopropylacrylamide) (PNIPAM), whose solubility in water decreases dramatically above 32 °C, are a promising option for the formulation of stimuli-sensitive nanocarriers. A sample of an amphiphilic diblock copolymer, consisting of PNIPAM and polymerized ionic liquid, appeared clearly monodisperse when analyzed at 25 °C by thermostatic AF4-MALS-RI. At 45 °C, on the other hand, a second population appeared as a consequence of phase transition. The copresence of two populations implies that phase transition is not complete at the temperature assayed [44].

The reactivity and binding properties of polymers can be assessed as well. Recent works employed AF4-UV to compare the binding affinity of different polyphosphazenes for several model proteins, vaccines antigens, and immune receptors. By measuring the quantity of free proteins, at different protein:polymer ratios, it was possible to estimate the maximum binding and the complexation constant of the polymers. In addition, a sharp increase in polyphosphazenes size, implying intermolecular aggregation, was caused by the proteins with the strongest affinities [105]. AF4-UV was also used to verify that the polyphosphazene-complexed E2 glycoprotein retained its antigenicity by incubating the complex with the HC84.26 antibody. The subsequent AF4 fractionation allowed to identify the formed E2-polyphosphazene-HC84.26 complex, proving that the glycoprotein was conformationally intact [106].

The interactions between fast-reacting samples can also be studied without previous incubation and instead using the fractionation channel as a reactor. The reagents are introduced in the channel by performing several injections in succession and left to react during the focusing step. The following fractionation step is used to separate the products from the reagent. Following this approach, AF4-UV-FLD served as the selection step in a SELEX cycle for the identification of aptamers for the enzyme DNA methyltransferases [107].

The characterization of proteins is another topic where AF4 can prove useful as quality control. Prolonged storage, freeze-thaw cycles, and thermal shocks are known to affect the polydispersity of the proteins and promote their aggregation, resulting in modification of their therapeutic properties. The flexibility in the choice of the eluent permits testing multiple conditions. For example, AF4-UV-MALS was applied to study the kinetics of thermal aggregation of albumin in presence of arginine derivatives [108] and the change in size of interferon-*τ* due to guanidine denaturation [109], while AF4-MALS-RI (RI: differential refractive index detection) to assess the increase in polydispersity of the tetanus toxin following its detoxification by formaldehyde treatment [110].

### Formulation studies: characterization of PEGylation and other polymeric shells

The most external layer of a nanocarrier has the double role of favoring the specific recognition at the targeted pathological site and avoiding the non-specific interactions that would lead to fast opsonization. This purpose can be achieved by adding an hydrophilic coating, such as PEG [111] or glycans functionalized or not [112]. Despite the importance of the PEG layer in balancing the stealth and targeting properties of the nanocarriers, its formation and stability are determined mostly in a qualitative or unprecise way [113]. The classic method contemplates quantifying the amount of unbound PEG present in the formulation via RI detection [114]. Similar methods can be applied to assess the density of different kinds of polymeric coatings.

A possible disadvantage of determining the extent of PEGylation by quantifying the unreacted polymer is that it does not allow to understand whether PEG forms a layer on the surface or it is trapped in the polymeric matrix [113]. The information obtained by AF4-MALS-RI, combined with other techniques such as small-angle X-ray scattering, can present a more complete picture of the system, helping in the determination of surface coverage and PEG chains orientation [115]. Another way to directly study the formation of the polymeric shell is to monitor the change in size of the particle as the coating polymer is added. In general, the amount of bound polymer can be accurately quantified only for high degrees of functionalization on small solutes of well-defined size, such as in the preparation of PEGylated asparaginase for the treatment of lymphoblastic leukemia and non-Hodgkin’s lymphoma [116]. This method of monitoring particle formation by measuring its mass or size before and after the addition of a reagent is sometimes called “differential FFF” [117, 118]. Differential FFF is perhaps more suited for SdFFF (v. list of references in [118]), thanks to its superior mass and size selectivity, although also AF4 can prove itself useful, especially for materials that lie below the operational size limits of SdFFF (*d* ≲ 100 nm) [119, 120]. Most of the examples reported, though, are proofs of concept carried out with highly monodisperse latex standards. For more polydisperse samples with less defined size, the uncertainty could be excessive, limiting the applicability of the differential FFF.

### Formulation studies: assessment of excess reagents in the formulation

Assessing the amount of the unreacted reagent present in the formulation becomes crucial when it can interfere with the activity of the drug. This is the case, for example, of the polycations, widely exploited as complexing agents for nucleic acids and which likely contribute to the endosomal escape via the proton sponge mechanism [121]. Their determination may be hindered by their strong positive charge, which often results in quantitative adsorption on the negatively charged membrane. To prevent sample loss, it may be required to work with a membrane with low surface charge and an eluent at pH close to its isoelectric point, as reported by Ma et al*.* for chitosan/DNA complexes [122, 123]. While these conditions are optimal for the detection of the polycations and the cationic nanoparticles via online UV-MALS-DLS detection, they result in the quantitative adsorption of the free nucleic acids on the membrane.

## Study the attachment of drug molecules to the nanocarriers and their release

### Determination of drug loading in polymeric nanocarriers

A primer for the determination of drug loading with AF4 is delineated in the classic review by Wagner et al. [12]. The amount of drug encapsulated in a carrier can be assessed directly, measuring the drug in the carriers, or indirectly, from the drug left free in the supernatant. AF4, coupled to suitable detectors, allows the determination of both the free and the encapsulated drug in a single run. Knowing how the drug is bound to the carrier is crucial for the choice of the right conditions, since weakly bound drugs could be washed out during the analysis. Small molecule drugs could be added to the eluent itself in order to keep their thermodynamic activity constant and avert their washing out, as exemplified in Venkatesh et al. [124] with SdFFF. In general, AF4 is not suitable for carriers whose release kinetics is on the timescale of minutes or is heavily concentration-dependent. In fact, it is known that in the AF4 channel, the samples are preconcentrated during the focusing step, which may force drug reassociation [125], and then greatly diluted by the main eluent flow when exiting the channel [126]. Since polymeric nanocarriers suffer sometimes from low incorporation stability and early drug release [127], the stability of the carrier during the separation is the first point to take into account to guarantee representative results of drug loading.

#### Choice of the detector

**Fig. 4:**
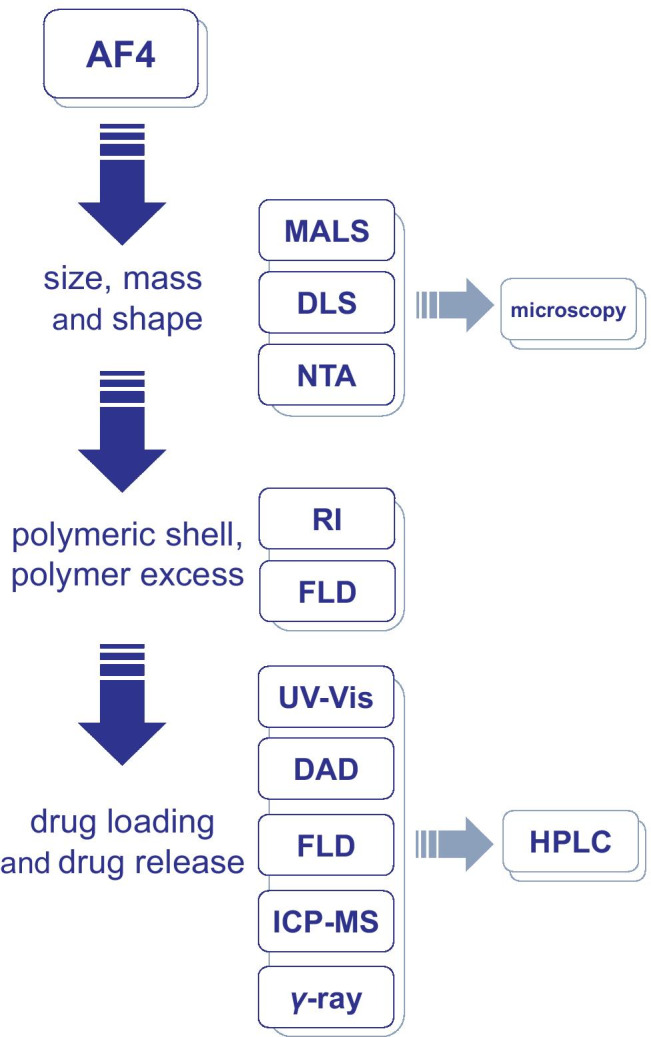
Hyphenation of AF4 to the appropriate detection system, both online and offline, allows in-depth characterization of the nanocarriers

Direct determination of the encapsulated drug is more accurate, but not always feasible. In many cases, the role of AF4 is limited to the separation of the carrier from the free drug, while the quantification is performed offline with a complementary technique such as HPLC [128–132]. Albeit of most general applicability, this approach is dispersive and time-consuming because of such issues as the need for preconcentrating the fractions and possible sample loss. As shown in Fig. 4, AF4 can be hyphenated to a wide variety of detectors, and their careful choice allows the online quantification of most types of drugs.

Drugs with strong chromophores, such as those used in photodynamic therapy, can be easily detected by UV absorption spectroscopy (UV/Vis) or fluorescence detection (FLD), even when encapsulated inside the nanocarriers [129, 131, 132]. The interference due to the anisotropic scattering could be a serious issue and should be evaluated by running samples of blank nanocarriers [133]. Direct detection is more suitable for drugs that adsorb at higher wavelengths—since light scattering is inversely related to the wavelength of the incident light—and for small nanocarriers, since scattering increases with particle size. In most cases, direct detection is best suited for fast screenings, while offline detection is recommended for more accurate analyses [129]. As one of the main advantages of AF4 is the ability to handle complex mixtures, a detection system capable of responding to different signals is important. Multichannel detectors (or diode-array detector, DAD) can prove useful for the simultaneous detection of multiple targets. In a study about cyclodextrins-based nanosystems, they allowed to detect at the same time the encapsulated clofazimine drug and the rhodamine-labeled cyclodextrins, both present in the same nanoassembly, by studying the variation of the absorption spectrum with the retention time [134].

For particular kinds of drugs, other detectors, albeit of narrower application, are available. Inductively coupled plasma-mass spectrometry (ICP-MS) is an elemental analysis technique commonly coupled to AF4 for the study of inorganic nanoparticles. But by targeting elements like sulfur, present in biological molecules in a known amount, it was successfully employed for the quantification of monoclonal antibodies, free and particle-conjugated [135–137]. Recently, it was reported the first example of a *γ*-ray detector coupled to AF4 for the quantification of liposomes-encapsulated ^212^Pb/^212^Bi radionuclides for cancer treatment [138]. *γ*-ray detection allowed reaching concentrations in the fmol range, well below the limits of detection of other elemental analysis techniques.

Online surface plasmon resonance (SPR) was also applied as an orthogonal method for the detection of the monoclonal antibody trastuzumab in serum samples. SPR is a specific and flexible label-free technique that can detect molecule-binding events in close proximity to the sensor surface. While the SPR sensor can specifically detect trastuzumab, the coupling with AF4 also allows to discriminate between monomers and aggregates of the monoclonal antibody. On the downside, SPR is limited by its own specificity, as it requires the immobilization of a suitable receptor (in this case, human epidermal growth factor receptor 2) on the surface of the sensor [139].

A summary of drug delivery systems, with the conditions and the detectors used to determine the drug loading in each case, can be found in Tables 3 and 4.

**Table 3:** Small-molecule drugs delivered by polymeric nanocarriers: conditions used for the fractionation and quantification of the associated drug

Drug	Nanocarrier	Eluent	Detector	Ref
^212^Pb-DTPA	DSPC/cholesterol/DSPE-DTPA liposomes	5 mM NaCl + 0.001% Tween-20	Online *γ*-ray detector	[138]
Aluminum chloride phthalocyanine	PLA nanocapsules, PEG-PLA nanocapsules, PLA-chitosan nanocapsules	MilliQ water	Offline HPLC-FLD	[131]
	PEG-P(LA-*co*-BGE) nanospheres	10 mM NaCl	Offline HPLC-FLD	[132]
Amphotericin B	Commercial liposomal formulation	0.5 mM NaCl	Offline HPLC–UV/VIS	[146]
Clofazimine	SBE-β-CD oligomers	MilliQ water	Online DAD UV/Vis	[134]
Curcumin	PMOXA-PDMS-PMOXA polymersomes	10 mM PB (pH 7.4)	Online FLD	[143]
	Star polymer micelles	1 × PBS (pH 7.4)	Online UV/VIS-FLD	[194]
Daunorubicin, docetaxel, doxorubicin, SN-38	Commercial micellar and liposomal formulations	1 × PBS	Offline HPLC–UV/VIS	[149]
Doxorubicin	Commercial liposomal formulation	100 mM NaCl	Offline HPLC–DAD	[148]
IR-780 Iodide	PEG-PLA nanocapsules	10 mM NaCl	Online FLD	[91]
Pheophorbide	PEO-b-PCL micelles and vesicles	0.02% NaN_3_	Online UV/Vis	[133]
*p*-THPP	Liposomes (E80S egg phospholipids)	10 mM TRIS (pH 7.4) + 0.02% NaN_3_	Online UV/Vis, offline HPLC–UV/Vis	[129]
Rose Bengal	Hyper-branched polymer decorated with maltose moieties	0.02% NaN_3_	Online UV/Vis (on the waste line)	[150]
Temoporfin (radioactively labeled)	POPC/POPG and DPPC/DPPG liposomes	0.02% NaN_3_	Offline scintillation counter	[145]

*DPPC* 1,2-dipalmitoyl-sn-glycero-3-phosphatidylcholine, *DPPG* 1,2-dipalmitoyl-sn-glycero-3-phosphatidylglycerol, *DSPC* 1,2-distearoyl-sn-glycero-3-phosphocholine, *DTPA* diethylenetriaminepentaacetic acid, *PEG* polyethylene glycol, *PEG-P*(LA-*co*-BGE) polyethylene glycol-block-poly-(D,L-lactide-co-benzyl glycidyl ether), *PEO-b-PCL* poly(ethyleneoxide-b-ε-caprolactone), *PLA* polylactic acid, *PMOXA-PDMS-PMOXA* poly(2-methyl-2-oxazoline)-block-poly-(dimethysiloxane)-block-poly(2-methyl-2-oxazoline), *POPC* 1-palmitoyl-2-oleoylphosphatidylcholine, *POPG* 1-palmitoyl-2-oleoyl-sn-glycero- 3-phosphatidylglycerol, *SBE-β-CD* sulfobutylether-*β*-ciclodextrins

**Table 4:** Proteins, peptides and nucleic acids delivered by polymeric nanocarriers: conditions used for the fractionation. All drugs were quantified by online UV/Vis spectrometry

	Drug	Nanocarrier	Eluent	Ref
Antibodies	Human IgG1-R, IgG1-T, murine IgG2a-A20 Id	Keyhole limpet hemocyanin conjugates	1 × PBS (pH 7.2) + 0.02% NaN_3_	[195]
Other proteins/peptides	Esterase (porcine liver), myoglobin (equine skeletal muscle)	PEG-DEAEMA-DMIBMA polymersomes	1 × PBS + 0.02% NaN_3_	[144]
	Peptide PCS5	Chitosan/dextran sulfate nanoparticles	SDS 0.1%	[38]
	Several proteins	Polyphosphazene (PCPP and PCEP) complexes	1 × PBS (pH 7.4)	[105]
	Lysozyme	Spermine-crosslinked polyphosphazene (PCPP and PCPP-PEG) nanoparticles	1 × PBS (pH 7.4)	[196]
	Nerve growth factor	PMOXA − PDMS − PMOXA polymersomes	10 mM PB (pH 7.4)	[143]
Nucleic acids and nucleosides	Fludarabine-5′-triphosphate, clofarabine-5′-triphosphate	Maltose-modified poly(propyleneimine) dendrimers	10 mM HEPES (pH 5.8 or 7.4)	[173]
	pGreen Lantern-1 plasmid	DOTAP/DOPE liposomes	89 mM Tris-borate (pH 8.59)	[197]
	Linear salmon sperm DNA, pEGFP-N1 plasmid	Hybrid micelles (PS-b-PQ2VP/oleic acid-stabilized iron oxide nanoparticles)	0.02% NaN_3_	[198]

*DOPE* 1,2-dioleoyl-sn-glycero-3-phosphoethanolamine, *DOTAP* 1,2-dioleoyl-3-trimethylammonium propane, *PCEP* poly[di(carboxylatoethylphenoxy)phosphazene], *PCPP* poly[di(carboxylatophenoxy)phosphazene, *PCPP-PEG* poly[di(carboxylatophenoxy)phosphazene-block-polyethylene glycol, *PEG-DEAEMA-DMIBMA* polyethylene glycol-block-diethylaminoethyl-methacrylate-block-4-(3,4-dimethylmaleicimido)butyl methacrylate, *PS-b-PQ2VP* polystyrene-blcok-poly(quaternized 2-vinylpyridine)

#### Drug localization in nanostructures

**Fig. 5:**
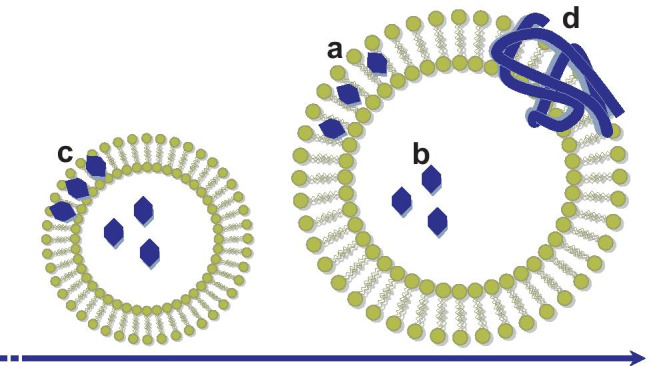
Drug location in vesicles, whether in the lipidic bilayer **a** or in the lumen **b** can be inferred by comparing drug distribution across vesicles of different sizes **c**. The inclusion of larger structures such as proteins may comport an increase in surface roughness **d**

AF4-MALS-DLS has been used for the characterization of vesicles and polymersomes because it can provide information about the location of the encapsulated drug, a major factor influencing its release. Depending on the characteristics of the drugs and on the loading method, the cargo can be located on the external surface, inside the membrane bilayer or in the core, as shown in Fig. 5. Empty spheres such as the polymersomes will have a shape factor of 1, while full spheres would have values closer to 0.778 [140]. Drugs molecules adsorbed on to the lipid bilayer would not cause a significant variation of the shape factor unless the adsorption promotes changes in the structure of the liposomes. In other cases, it was reported that vesicle-peptide interactions lead to an increase in vesicle size, while their shape factor remained equal to 1 [141]. A similar increase in size, but not in polydispersity nor shape factor, was found after the inclusion of the hydrophobic drug paclitaxel within the lipids bilayer of multilamellar vesicles [142]. However, drug inclusion within the bilayer does not always result in visible changes of the properties of the vesicles [143].

While the main factor in determining the location of small drugs is their lipophilicity, the case of larger, more complex structures such as proteins is much less straightforward. A recent example regarded the post-loading of proteins of different sizes (myoglobin, 5 nm, and esterase, 10 nm) into polymersomes [144]. Although the location of other types of cargo, such as Au nanoparticles, can be visually assessed by electronic microscopy, this is not the case of proteins. AF4-MALS-DLS could assess that protein incorporation does not affect the size of the vesicles size, but it provokes an increase of the membrane surface roughness, as suggested by the analysis of the fractal dimension and shape factor. The conclusion was that the proteins are mostly located in the membrane region. Such an increase in roughness was more consistent for esterase, suggesting that myoglobin was able to penetrate the membrane while esterase remains adsorbed on the outer layer, causing a more apparent deformation of the surface.

#### Drug localization in polydispersed populations of nanostructures

Several works studied how additives such as cholesterol and drugs such as temoporfin and amphotericin B partition over a population of polydispersed liposomes [145, 146]. It was found that, while the loading of cholesterol is size-independent, the loading of temoporfin and amphotericin B increases linearly with the size of the liposomes. It is known that amphotericin B strongly interacts with the sterols present in biological membranes, sequestrating them from the lipid phase and forming sponge-like structures on the membrane surface [147]. The formation and growth of said structures depend on the curvature of the membrane, and it increases with particle size. A similar study has been carried out on a liposomal formulation of doxorubicin [148]. In this case, thanks to AF4 and offline HPLC analysis, it was possible to demonstrate that since doxorubicin is hydrophilic and is loaded inside the water core, the drug loading was independent of the membrane curvature. Again, a liposomal formulation composed of three subpopulations was loaded with both doxorubicin and docetaxel. Offline HPLC analysis showed that the loading of the two drugs was related not only to the liposome size but also to their location inside the vesicle. The hydrophobic docetaxel, located in the lipid bilayer, was more prone to an early release from the carrier than doxorubicin, loaded in the lumen [149].

#### Detection of small-molecule drugs

A limitation of AF4 is that it cannot fractionate materials of sub-nanometric size, as they will easily slip through the pores of the membrane. Most small-molecule drugs, if not bound to their carrier, will then get lost, posing a problem to drug loading or drug release studies. A possible solution could be to have them interact with components of the eluent—e.g., detergent—to form larger complexes that remain inside the channel [38]. Though interesting, this solution clearly is not universally applicable. A possible alternative could be to recover the eluent filtrated through the membrane and quantify the amount of filtrated sample [150]. Sample loss on the membrane could be a major issue for quantitative analysis, and several sacrificial injections of the drug must be performed to saturate the membrane and avoid non-specific adsorption. Although promising, this application of AF4 as a filtration device met with limited success and so far has been applied mostly to the separation of dissolved metal ions, from Ag nanoparticles [151] or, more recently, from dissolved organic matter in lake water samples [152].

### Evaluation of drug release from polymeric nanocarriers

The kinetics of drug release is one of the primary parameters that define the efficiency of a drug formulation. This is normally studied through in vitro release tests, in which the nanoformulation is dispersed in a release medium at physiological temperature, and the amount of drug released to the medium is then monitored as a function of time. In the traditional “sample and separate methods,” aliquots of the dispersions are collected at different times and subjected to suitable separation techniques in order to isolate the drug from the carrier [153]. Unfortunately, several of the techniques used for this purpose, such as filtration and centrifugation, may degrade the carriers or promote drug release [154]. Dialysis-based methods present the advantage of being much softer and allow online detection, although they are not suitable for rapidly released drugs, as membrane transfer itself may become rate-limiting [155].

#### Release of lipophilic drugs

The aforementioned techniques, though, show reduced predictive power in the case of lipophilic drugs, where the main factor affecting the kinetics of drug release in vitro is the solubility of the drug in the release medium. This is overcome by working in a biphasic system such as oil droplets or a surfactant-enriched medium capable of dissolving the hydrophobic drug (“External Sink methods”) [156]. Nevertheless, sampling the release medium requires a suitable separation method, which poses the same disadvantages cited before [157]. A series of articles [129, 158–160] demonstrated how AF4 can perform drug release and drug transfer studies by combining the advantages of the classic Sample and Separate method with the External Sink method. In particular, AF4-UV allows online drug content quantification and is not limited by the size or properties of the drug or the carrier. The system considered was constituted by liposomes loaded with a model drug, the photosensitizer meso-tetra(hydroxyphenyl)porphyrin (*p*-THPP), which were incubated together with much larger vesicles. Samples taken from the dispersion were subsequently fractionated to determine the amount of drug present in the liposomes or in the larger vesicles as a function of time. Drug transfer can be followed by monitoring online the decrease of the drug concentration in the liposomes, or by offline UV detection of both the donor and acceptor fractions. This last option also allowed to estimate the amount of drug lost in the aqueous phase. The size of the acceptor vesicles should be optimized in order to guarantee a reasonable baseline separation from the donor liposomes and reasonably short elution times [129]. The optimized method was employed to study the kinetics of drug release of several lipophilic drugs and dyes, for different ratios of acceptor and donor vesicles, for mono- and multi-lamellar vesicles [160].

#### Drug transfer to plasma proteins

**Fig. 6:**
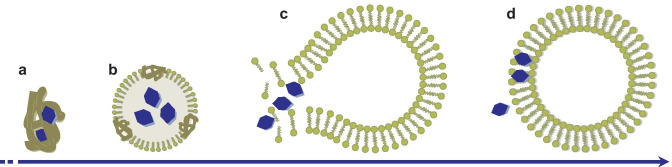
Being able to fractionate complex mixtures, AF4 can characterize the transfer of small-molecule drugs to lipophilic scavengers such as albumin **a** or lipoproteins **b**. AF4 can also help to differentiate whether the release happens as a consequence of carrier disruption **c** or drug diffusion **d**

Nanocarriers in the bloodstream interact with a plethora of biomolecules that may result in premature drug release and sequestrate the free drug, with drug-protein binding being one of the principal factors affecting drug release [161]. Most of the techniques commonly applied to study it, such as dialysis, filtration, or centrifugation [162], may not be able to separate efficiently the nanocarriers from the proteins, leading to an underestimation of the release rate. As exemplified in Fig. 6, AF4 can be particularly useful in these situations, being an ideal technique when it comes to fractionating complex mixtures. Numerous methods have been developed for the analysis of complete plasma or serum and can be easily applied to study drug transfer to plasma proteins [163–167]. As a common rule, plasma samples are fractionated in physiological buffers to preserve the structure and properties of the proteins. Detergents [163] or other suitable reagents such as imidazole [165] can be added to prevent non-specific interactions with the channel walls or the membrane. The identity of the proteins can be guessed by studying their size and shape factor as determined by online MALS [165] or by running protein standards [163, 164] or, for greater accuracy, by adding a selective stain [168] or by performing HPLC-MS analyses offline [167]. The proteins with which the drug interacts can be identified by downstream analyses of the collected fractions. Further treatment may have to be carried out on the fractions before the analysis, depending on the drug, its concentration, and the nature of drug-protein binding. For example, the antineoplastic midostaurin could be directly quantified by fluorescence spectroscopy, as albumin, the protein with which it mainly interacts, did not interfere with the detection [163]. On the other hand, when studying the distribution of different kinds of aptamers in serum, the fractions had to be subjected to bead-based extraction and subsequent amplification by polymerase chain reaction [50]. If the drug is labeled, its interactions with proteins can be studied online, by observing how their peak shift at longer times or new populations appear after incubation in plasma [166, 169]. AF4 presents several limitations, though: peak broadening is more accentuated than chromatography, and baseline separation is often unfeasible. Although deconvolution methods have been developed to improve peak resolution [170], more often than not it may not be possible to identify exactly the protein to which the drug is transferred: consider, for example, that albumin and high-density lipoproteins are both acceptors of lipophilic drugs, and being of similar size their peaks have a high chance of overlapping [164, 167].

Once acknowledged its limitations, AF4 is a powerful tool in the study of drug release to serum proteins. In a recent case, polylactic acid nanocapsules were loaded with a fluorescent probe, both by covalently linking the probe to the polymer and via encapsulation inside the oily core. The fluorescently labeled nanocapsules were incubated in serum and directly injected in the AF4-UV-FLD-MALS-DLS system. Only when using a covalent link, the carriers retained their fluorescence after the incubation, while nearly 50% of the physically encapsulated probe was transferred to the proteins after less than one hour of incubation. It is noteworthy that in the absence of serum proteins, the nanocapsules did not release the encapsulated probe during the fractionation, demonstrating that it was not simply washed out as a consequence of dilution [169].

More sophisticated analyses can be carried out on the appropriate systems. Liposomes incubated in plasma can rapidly transfer lipophilic drugs such as temoporfin to lipoproteins, and in a series of works [168, 171] AF4 was employed to investigate the mechanism of said transfer: whether it happens through direct diffusion from the liposomes to the lipoproteins adsorbed on the vesicle surface, or because the vesicles membrane disintegrates. A lipid radiolabel was added to the temoporfin-loaded liposomes. AF4 was then used to isolate the fraction corresponding to the liposomes, whose temoporfin and lipid content was measured offline. While drug diffusion would result in a decrease of the drug/lipid ratio in the liposome fraction, the ratio would stay constant if both the drug and the lipid were released because of vesicle disruption. With this method the authors were able to correlate the mechanism of drug transfer to the kind of phospholipids, their degree of PEGylation, the presence of cholesterol in the vesicle membrane, and the drug loading. Information about particle stability and drug release obtained from AF4 can be a good predictor of the behavior of nanocarriers after intravenous administration and could be used as a pre-screening to select formulations suitable for in-vivo testing [172].

#### Fractionation and kinetics of drug release

In general, before deciding whether AF4 is suitable or not for the study of drug release, it is important to have an estimate of the kinetics of the overall process. A complete fractionation could last up to 1 h, the same timescale of some drug-transfer processes. It must be remembered though that if donors and acceptors have sufficiently different sizes, drug transfer is highly unlikely to take place during the fractionation itself, as the particles will be set too far apart in the channel. Conversely, drug transfer could be physically possible during the focusing step, where donors and acceptors are pressed on each other by the cross-flow; this is compensated by the fact that the timescale of the focusing is much shorter (4–10 min) [129]. Especially in cases when drug release is medium-sensitive, the composition of the eluent should match the release medium, to prevent undesired drug release or reassociation [173].

## Study the interactions with biomolecules and protein corona

Nanomaterials in biological fluids instantaneously interact with an excess of biomolecules that adsorb on their surface, which can radically alter their properties and biological fate. Nanoparticle-associated proteins can be empirically classified as belonging to a “hard corona” of proteins firmly adsorbed onto the particles and a “soft corona” of proteins loosely bound and rapidly exchanging with the surroundings. The biocorona is a dynamic system and separating the particles from the biological fluid while preserving the integrity of the corona can be arduous. Centrifugation, coupled with several washing steps, is usually the method of choice. Common problems experienced in the separation are the contamination from free plasma proteins, the desorption of the bound proteins, and the disruption of the nanoparticles. This last issue could be particularly serious with some polymeric nanocarriers (e.g., liposomes) due to their fragility. Chromatographic methods, such as capillary electrophoresis and AF4, have been proposed as befitting alternatives [174, 175].

The application of AF4 to the study of biocorona is twofold: on the one hand, differential FFF can be applied to study corona formation by monitoring differences in size distribution before and after the incubation in biological media [91, 176]. On the other hand, it can serve as a fractionation technique that precedes the ex situ proteomic analysis, carried out by other means (electrophoresis, mass spectrometry). Most FFF-based studies of protein corona composition regard solid nanoparticles, such as iron oxide nanoparticles [177, 178], Ag nanoparticles [179–181], or latex [182], but a few novel studies have been devoted to polymeric nanocarriers [183, 184].

### Corona thickness

When differential FFF is applied, high sample recovery and ease of detection are the primary objectives, and non-physiological conditions are sometimes needed for unstable particles: for example, detergent mixtures (iron oxide nanoparticles in rat blood [185], Ag nanoparticles and albumin [186]), or strong basic conditions (Ag nanoparticles in synthetic plasma [180]). As a sizing technique AF4 presents the clear advantage over DLS that it rules out any apparent increase in size due to the presence of larger aggregates in the medium [187]. Protein corona on polymeric systems tend to be heterogeneous and only a few nanometers thick [188], and corona thickness could be simply too small to be precisely assessed, especially in hydrophilic systems [39, 187]. Moreover, proteins and nanoparticles of similar size but different conformation may co-elute, interfering in the size determination by light scattering. A real change in size should be accompanied by some variation in the retention time [176]. As stated above, SdFFF, thanks to its superior resolution, can be a more suitable technique than AF4. Differential SdFFF-UV-DLS was able to detect the formation of an antibody layer as thin as 7–8 nm on polystyrene nanoparticles with a diameter of 100–196 nm [118].

### Corona composition

When AF4 is preliminary to proteomic analysis, the preservation of the integrity of the corona is of paramount importance, in addition to respecting the conformation and properties of the proteins. The eluent needs to be chosen so as to match the incubation medium, devoid of the macromolecular fraction: for nanoparticles incubated in plasma or serum, PBS or phosphate buffers (PB) are standard choices for the eluent [39, 182, 183, 189], while protein-free intestinal fluid is an option for orally administered drugs [190]. As mentioned above, fractionation carried out with sufficiently mild separation techniques may lead to the retrieval of the corona in its entirety, while harsher conditions would cause the most loosely bound proteins to be washed away. The applications of multiple techniques may thus help to discern which proteins belong to the hard and soft corona. The few studies that systematically compared the performance of centrifugation and AF4 lead to ambiguous results, regarding which of the two techniques can preserve the corona in its pristine conditions. Actually, there is evidence supporting both claims: while AF4 operates in much milder conditions than centrifugation and it is more adapt to fragile systems [182, 183, 189], it is true that proteins with very fast dissociation rate could be washed off by the flow preventing their re-association to the particles [177–179].

A source of uncertainty in the identification of the proteins belonging to the corona is given by the possible coelution of free proteins with the nanoparticles. Most nanocarriers are larger than the majoritarian plasma proteins. Low-density and very-low density lipoproteins, though, have a size comparable to most of the nanoparticles used in drug delivery. This can be particularly serious for apolipoproteins, as they are known to be present in the corona of most polymeric nanoparticles [168]. Further interference can be due to aggregates of smaller proteins, both originated by the freeze-thawing of plasma samples and naturally present. In order to discriminate between the proteins of the corona and the free ones, a proposed strategy consists of adding a protein-labeling agent to the fractions obtained by AF4, and then analyze them by flow-cytometry [182, 183]. In this way, the fluorescently labeled proteins are detectable only when bound to the particles, as the free proteins lie below the size limit of the cytometer. In spite of this, the presence of false positives is a serious issue, deserving careful attention. A newer study [184] rightly recommends a more scrupulous protocol: controls of both plasma and nanoparticles alone are run in the same conditions as the plasma-incubated nanoparticles. Then, a label-free quantitative proteomic analysis identifies the proteins in the incubated nanoparticles that are upregulated with respect to the two controls. Only the proteins upregulated with respect to each of them can be safely assumed to belong to the corona, while the others will be contaminants already present on the particles or co-eluting free plasma proteins. These stricter conditions were imposed on the study of core-cross-linked nanoparticles with a hydrophilic coating. As a result, the amount of proteins that could be positively attributed to the corona became practically non-existent, with the vast majority of nanoparticles not being associated to any protein. This could both imply that the nanosystems examined are effectively resistant to protein corona formation thanks to their hydrophilic coating, or that the proteins are only weakly bound and dissociate during the analysis.

To conclude, without a previous idea of the dissociation kinetics of the proteins, it is not possible to predict a priori whether the corona would withstand the fractionation or not.

## Conclusions

Asymmetric flow FFF is the most popular and easily implementable FFF technique among those available on the market. If compared with other separation techniques, though, it is still in its infancy and its potential is often underestimated. In this primer, we tried to give a comprehensive review of the kinds of information, meaningful to the pharmaceutical technologist, that can be retrieved by AF4.

Aside from particle size, when coupled to suitable detectors, AF4 can provide information about the efficiency of a formulation process and the presence of unreacted reagents and subpopulations. In the case of polydisperse formulations, AF4 can investigate the correlation between drug loading and nanocarrier size distribution, a sort of information that can be difficultly obtained with other techniques. The effect of biological fluids on nanocarrier stability and drug-release can be studied as well. The lack of a sieve or stationary phase set AF4 apart from other chromatographic and filtration techniques and allows working with more broadly dispersed samples, a crucial advantage when it comes to separating molecular drugs or small proteins from nanocarriers, and nanocarriers from particles of micrometric size. We must stress that AF4 does not substitute other fractionation techniques, but it complements them.

The pitfalls of this technique, that still restrained it from being more widely applied in the field of pharmaceutics, are acknowledged as well. In particular, we would like to draw the reader’s attention to two topics that deserve thorough consideration, by experimentalists and theorists alike: first, the issue of the sample-membrane interactions would derive substantial benefit from an extensive characterization of the membranes available on the market. Second, we recommend addressing the effects of fractionation and dilution on the dissociation kinetics of complexes. This would contribute to explain whether, or under which circumstances, AF4 is suitable to the study of labile interactions, such as those between the nanomaterials and the biomolecules that constitute the soft biocorona or the characterization of particularly fast-releasing nanocarriers.
