# Correction to: Asymmetric flow field‑flow fractionation as a multifunctional technique for the characterization of polymeric nanocarriers

**DOI:** 10.1007/s13346-021-00962-1

**Published:** 2021-03-19

**Authors:** Federico Quattrini, Germán Berrecoso, José Crecente‑Campo, María José Alonso

**Affiliations:** 1 Center for Research in Molecular Medicine and Chronic Diseases, Singular Research Centers, 15782 Santiago de Compostela, Spain; 2 Instituto de Investigación Sanitaria de Santiago de Compostela (IDIS), IDIS Research Institute, 15706 Santiago de Compostela, Spain; 3 Department of Pharmacy and Pharmaceutical Technology, School of Pharmacy, Universidade de Santiago de Compostela, 15782 Santiago de Compostela, Spain

## Correction to: Drug Delivery and Translational Research 10.1007/s13346-021-00918-5

   The article Asymmetric flow field‑flow fractionation as a multifunctional technique for the characterization of polymeric nanocarriers, written by Quattrini et al., was originally published electronically on the publisher’s internet portal on January 31, 2021, without open access. With the authors’ decision to opt for Open Choice the copyright of the article changed on March 3, 2021, to ©The Author(s) and the article is forthwith distributed under a Creative Commons Attribution 4.0 International License.

